# The Italian National Registry for FSHD: an enhanced data integration and an analytics framework towards Smart Health Care and Precision Medicine for a rare disease

**DOI:** 10.1186/s13023-021-02100-z

**Published:** 2021-11-04

**Authors:** Cinzia Bettio, Valentina Salsi, Mirko Orsini, Enrico Calanchi, Luca Magnotta, Luca Gagliardelli, June Kinoshita, Sonia Bergamaschi, Rossella Tupler

**Affiliations:** 1 Department of Biomedical, Metabolic and Neural Sciences, University of Modena and Reggio Emilia, Modena, Italy; 2 Department of Life Sciences, University of Modena and Reggio Emilia, Modena, Italy; 3 DataRiver Srl, Modena, Italy; 4 Department of Engineering “Enzo Ferrari”, University of Modena and Reggio Emilia, Modena, Italy; 5 FSHD Society, Reading, MA USA; 6 Department of Molecular Cell and Cancer Biology, University of Massachusetts Medical School, Worcester, USA

**Keywords:** Rare disease registry, Data collection, Data integration, FSHD, Rare diseases

## Abstract

**Background:**

The *Italian Clinical network for FSHD* (ICNF) has established the Italian National Registry for FSHD (INRF), collecting data from patients affected by Facioscapulohumeral dystrophy (FSHD) and their relatives. The INRF has gathered data from molecular analysis, clinical evaluation, anamnestic information, and family history from more than 3500 participants.

**Methods:**

A data management framework, called *Mediator Environment for Multiple Information Sources* (MOMIS) FSHD Web Platform, has been developed to provide charts, maps and search tools customized for specific needs. Patients’ samples and their clinical information derives from the *Italian Clinical network for FSHD* (ICNF), a consortium consisting of fourteen neuromuscular clinics distributed across Italy. The tools used to collect, integrate, and visualize clinical, molecular and natural history information about patients affected by FSHD and their relatives are described.

**Results:**

The INRF collected the molecular data regarding FSHD diagnosis conducted on 7197 subjects and identified 3362 individuals carrying a *D4Z4 Reduced Allele* (DRA): 1634 were unrelated index cases. In 1032 cases the molecular testing has been extended to 3747 relatives, 1728 carrying a DRA. Since 2009 molecular analysis has been accompanied by clinical evaluation based standardized evaluation protocols. In the period 2009–2020, 3577 clinical forms have been collected, 2059 follow the Comprehensive Clinical Evaluation form (CCEF). The integration of standardized clinical information and molecular data has made possible to demonstrate the wide phenotypic variability of FSHD. The MOMIS (Mediator Environment for Multiple Information Sources) data integration framework allowed performing genotype–phenotype correlation studies, and generated information of medical importance either for clinical practice or genetic counseling.

**Conclusion:**

The platform implemented for the FSHD Registry data collection based on OpenClinica meets the requirement to integrate patient/disease information, as well as the need to adapt dynamically to security and privacy concerns. Our results indicate that the quality of data collection in a multi-integrated approach is fundamental for clinical and epidemiological research in a rare disease and may have great value in allowing us to redefine diagnostic criteria and disease markers for FSHD. By extending the use of the MOMIS data integration framework to other countries and the longitudinal systematic collection of standardized clinical data will facilitate the understanding of disease natural history and offer valuable inputs towards trial readiness. This approach is of high significance to FSHD medical community and also to rare disease research in general.

**Supplementary Information:**

The online version contains supplementary material available at 10.1186/s13023-021-02100-z.

## Introduction

Facioscapulohumeral muscular dystrophy (FSHD) (MIM#158900), the third most common hereditary myopathy with prevalence of 1 in 20,000 [1], is characterized by progressive and variable atrophy and weakness of the facial, shoulder, and upper-arm muscles [2]. Wide variability of the clinical spectrum ranging from asymptomatic subjects to patients who are wheelchair dependent has been described among and within FSHD families [3]. Before the advent of molecular diagnosis, penetrance of FSHD was evaluated on clinical examination of patients at risk and estimated between 83 and 95% by the age of 20 years [4]. FSHD is considered an autosomal dominant disorder, associated with rearrangements occurring in a 3.3 kilobase (kb) tandemly repeated sequence (D4Z4) located at the 4q subtelomere [5]. The analysis of FSHD penetrance [3, 6–14] reinforced the idea that additional elements take part in disease onset and progression. Indeed, the clinical variability and non-penetrance observed in presence of the same molecular defect affect diagnosis, prognosis, genetic counseling. Besides having a great impact in clinical practice, the observed phenotypic heterogeneity is also affecting research and readiness to clinical trials. To address these issues, the Italian Clinical Network for FSHD (ICNF) developed clinical tools, the FSHD scale (that computes the FSHD score [15], and the Comprehensive Clinical Evaluation Form (CCEF) [16], for the standardized evaluation of subjects. Based on the observed phenotypic features, one individual can be assigned to one of four different phenotypic categories (A, B, C, D). To integrate molecular data with clinical data the Italian National Consortium for FSHD (INCF) established the Italian National Registry for FSHD (INRF). The INRF has accrued 3362 subjects carrying *D4Z4 Reduced Alleles* (DRA), including 1032 unrelated families with 1728 relatives heterozygotes for a DRA. This large collection of data, the largest world-wide, has enormous potential for providing clinical and epidemiological information and requires suitable tools for proper collection, storage and analysis.

In 2016, a collaboration between the INCF and DataRiver S.r.l. realized the *MOMIS FSHD Web Platform*. It consists of two main web modules, based on the open-source EDC platform OpenClinica and the MOMIS Data Integration System for data search, analysis, and extraction. The integration of multiple distributed data sources with genomic information, family origin and instrumental investigations will result in a greater depth and fast growth of information that will be managed. The MOMIS FSHD Web Platform is an open framework to enable collaboration and knowledge exchange for biomedical and clinical research. All information from different sources is integrated to constitute a solid support for clinical practice and research on the disease.

Here we describe the organization and summarize the results obtained from the implementation of the *MOMIS FSHD Web Platform*.

## Methods

### MOMIS framework architecture

The MOMIS FSHD Web Platform integrates data coming from different, heterogeneous and fragmented data sources. The platform provides a unified vision of integrated clinical data to facilitate data exploration and analytics through the MOMIS data integration system.

**Fig. 1:**
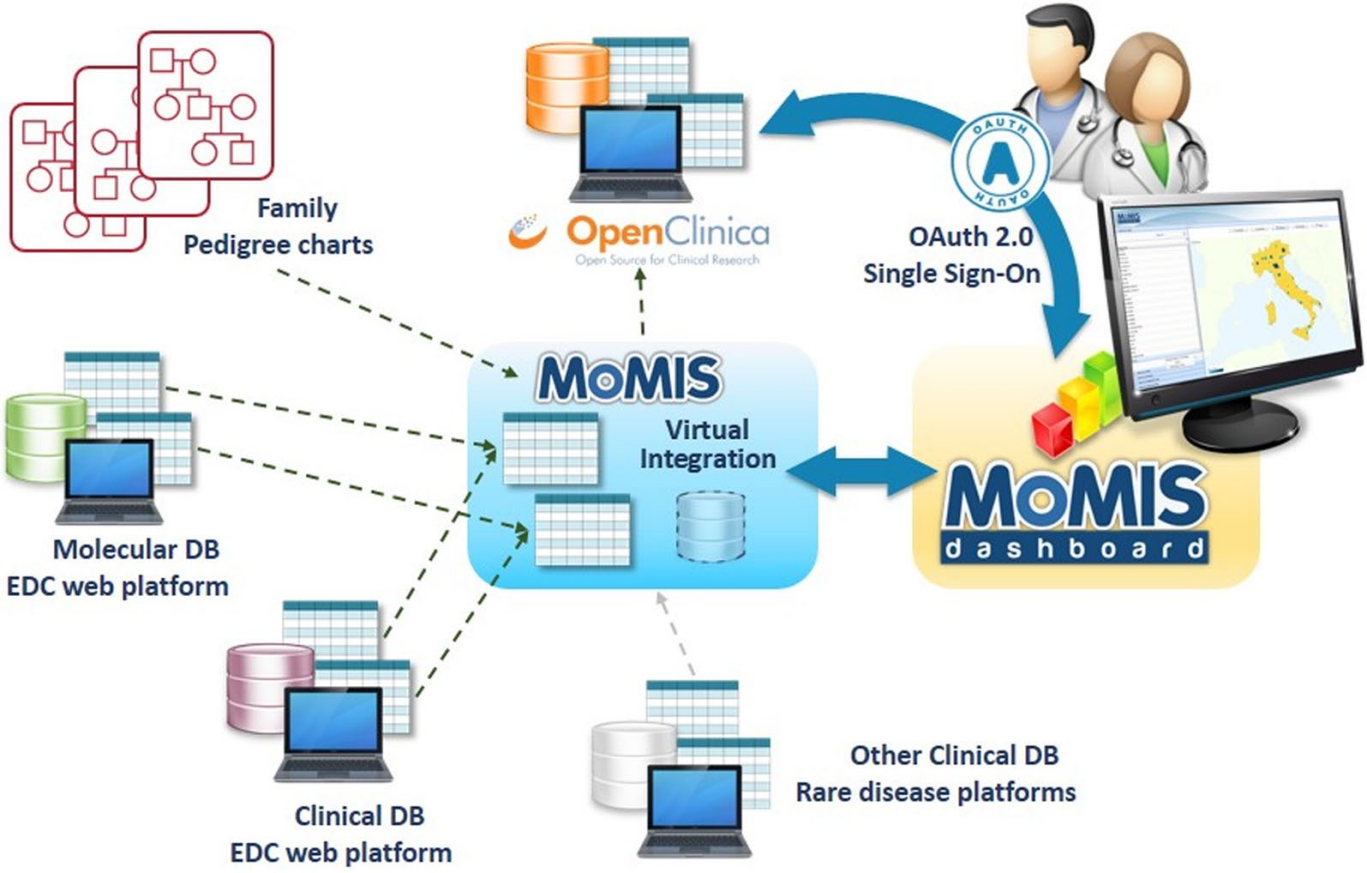
Architecture of the INRF based on the MOMIS FSHD Web Platform

Figure 1 shows the architecture of the FSHD Registry. Integrated data came from three different sources:

- *Family Pedigree Charts* reporting the family pedigree of each index case;
- *Molecular DB web platform* contains molecular analysis data of patients and their relatives;
- *Clinical DB web platform* contains clinical data of patients and their relatives.

For the integration of these three data sources, we used the MOMIS (Mediator Environment for Multiple Information Sources) data integration framework designed by the DBGROUP at the University of Modena & Reggio Emilia and developed by DataRiver. MOMIS builds a virtual unified schema, which preserves the independence and security of original data sources allowing users to formulate queries on it. The integration process consists of four main steps: (i) connection sources and automatic extraction of schema of the selected data sources; (ii) automatic annotation of the schemas attributes, i.e., one or more meanings is associated to each of them; (iii) attributes with the same meanings are automatically connected by semantic mappings; (iv) finally, a global schema is generated and can be manually refined by the data integrator designer.

MOMIS has already been used in several integration projects [17], including clinical trials and public authorities data management [18]. The MOMIS FSHD Web Platform relies on OpenClinica™ Community Edition (Fig. 1) to manage Clinical Data information, strengthening data quality and efficiency. The data collected through OpenClinica are displayed through the MOMIS Dashboard [17, 18]: a MOMIS framework’s web tool for data analysis and monitoring tool realized by DataRiver (Fig. 1).

### OpenClinica database

The INRF Database collects clinical and molecular data from both index cases and their relatives.

To manage an auditable collection of clinical data we used the open-source web application OpenClinica™ Community Edition. The OpenClinica Electronic Data Capture (EDC) system is a widely used and well-trusted clinical research web platform used for all types of clinical studies, population and rare disease registries. It is currently used by hundreds of research centers and institutions in over 100 countries and can rely on a community of about 18,000 users. The platform is compliant with NIH, HIPAA, 21 CFR Part 11 requirements and regulatory guidelines for clinical data management [19]. OpenClinica is designed as a standards-based, extensible, and modular platform, to guarantee the absolute and strict compliance with the confidentiality and security constraints of access to highly sensitive data.

The OpenClinica platform has been extended with the EDC module to provide the user with specific features to manage sample providers, families, probands and pedigrees. Blood relationships between subjects are crucial to study in detail the families and sporadic cases in which there is an identified molecular defect typical of FSHD. The customized software modules allow users to manage family relationships, identifying the proband (the first family subject affected by disease) and streamlining searches on family branches that could be far from their geographical origin. Through a secure connection, the EDC module allows users to easily switch between the MOMIS Dashboard search and monitoring tool; the custom software modules to manage families, probands and subjects; the data capture forms implemented on OpenClinica.

### MOMIS data processing workflow

When the sample of a new patient arrives at the Miogen Lab, the MOMIS FSHD Web Platform provides a new and unique identifier in the format ID/YEAR. The system then creates two new *Events* in OpenClinica: “Registration” and “Visit”. The *Registration* form is designed to collect the participant’s personal data and other information concerning the arrival day of the sample, the National Health System request, and the informed consent. The visit event includes the *Molecular* and the *Comprehensive Clinical Evaluation Form* (CCEF) modules. Case Report Forms (CRFs) are designed to accurately collect all this information from each subject investigated by the ICNF. This allows performing a solid epidemiologic data analysis based on the study of families and sporadic cases.

The modularity, the flexibility, and the ability to create custom forms and fields made the platform a very suitable resource to accomplish our purposes.

### The MOMIS Dashboard in the clinical practice of FSHD

1. The molecular module

The Molecular module is used for tracking the sample in each step of molecular analyses (e.g., the identifiers of the autoradiographs showing the results of molecular investigation, or the sample storage location). As the final step of the molecular analysis, the allelic profile of the patient is reported. The molecular report contains the size of each D4Z4 allele at specific loci (i.e., Chr-4 q35 or Chr-10 q26), alleles of the qA/qB polymorphism. Additional molecular information such as the presence of a *polyadenylation signal* (PAS) and/or *simple sequence length polymorphism* (SSLP) or D4Z4 methylation status can be uploaded. It is also possible to register additional molecular information such as Next Generation Sequencing data.

2. The comprehensive clinical evaluation form

Medical experience and systematic clinical evaluation of hundreds of participants guided the evolution of the clinical form from the original FSHD Clinical Form [15], to the *Comprehensive Clinical Evaluation Form* (CCEF) [16], which collects several additional information such as comorbidities, common and uncommon signs of the disease, lifestyle habits, and more importantly the clinical categories. The CCEF has been validated and frozen in 2016 [16]. The CCEF is organized in four sections as summarized in Table 1.

**Table 1:** Description of the main features in the COMPREHENSIVE CLINICAL EVALUATION FORM (CCEF)

Section 1: The evaluation form	Section 2: FSHD evaluation scale and FSHD clinical score	Section 3: Clinical diagnostic form	Section 4: Clinical category assessment
Evaluation form collects several information and is subdivided in three part: *Part A* Investigates the subject’s clinical history *Part B* Evaluates the patient’s disability (neurological examination) *Part C* Assesses muscle segmental involvement using the *Medical Research Council* (MRC) scale	Evaluation scale allows the computation of the FSHD SCORE which is based on the regional distribution of muscle weakness and the functionality of muscle groups Five groups of muscles are investigated and evaluated by a specific score: 1 *facial muscles* (scored from 0 to 2) 2 *scapular girdle muscles* (scored from 0 to 3) 3 *upper limb muscles* (scored from 0 to 2) *leg muscles* (scored from 0 to 2) 4 *pelvic girdle muscles* (scored from 0 to 5) 5 *abdominal muscles* (scored from 0 to 1) The FSHD score ranges from 0 to 15 and indicates the severity of the motor impairment	This section established the assessment of typical and atypical features of FSHD An accurate evaluation of these features allows to assign clinical categories	Assignment to each individual one out of four different phenotypic categories: *typical FSHD:* category A, subcategories A1, A2, A3 *incomplete phenotypes:* category B, subcategories B1, B2 *asymptomatic/healthy subjects*: category C, subcategories C1, C2 *atypical/complex phenotypes:* category D, subcategories D1, D2

The *FSHD Evaluation Scale* is used to calculate the FSHD clinical score based on the regional distribution of muscle weakness and the functionality of muscle groups. The FSHD score ranges from 0 to 15 and indicates the severity of the motor impairment.

The *Clinical Diagnostic section* is used to report the combination of typical or uncommon clinical features of FSHD.

The section 4, *Clinical Categories,* assigns the individual to one out of four different phenotypic categories: typical FSHD (category A, subcategories A1, A2, A3), incomplete phenotypes (category B, subcategories B1, B2), asymptomatic/healthy subjects (category C, subcategories C1, C2) and atypical/complex phenotypes (category D, subcategories D1, D2).

The development of the Molecular report and the CCEF in OpenClinica has generated unprecedented possibilities towards the understanding of the FSHD disease. The collection and management of numeric and objective data regarding patients and families makes the search for patterns and correlations not only feasible but also easy.

3. The filtering module

In clinical practice, the MOMIS FSHD Web Platform can be used to obtain an integrated view of data coming from several commercial DBMSs. The graphical interface of MOMIS Dashboard allows a quick and effective data extraction from the database. Since 2007, the Italian Registry for FSHD has collected clinical data coming from 3577 subjects (3362 DRA carriers). For a first cursory analysis subjects can be grouped, classified, and filtered for one or more features. For this reason, the MOMIS FSHD Web Platform’s homepage provides different features to filter the overall database population and to extract valuable information in the Microsoft Excel Spreadsheet format.

The filter options include:

1. **Subject:** this option filters by subject ID, name, surname, restricting the search to only ‘probands’ (index cases of unrelated families). It is also possible to select only subjects who received a FSHD Score (an index of degree of muscle impairment), within a configurable range.
2. **Category**: it is possible to filter by the phenotypic clinical category. On the basis of the observed phenotypic features, one individual can be assigned to one out of four different phenotypic clinical categories: typical FSHD (category A, subcategories A1, A2, A3), incomplete phenotypes (category B, subcategories B1, B2), asymptomatic/healthy subjects (category C, subcategories C1, C2) and atypical/complex phenotypes (category D, subcategories D1, D2) [15].
3. **Family**: filters by family name.
4. **Registration Year**: the year of patient registration in the database can be selected.
5. **Alleles**: filters by the characteristics of the monitored alleles, e.g., encountered peculiarities, type of translocation and allele size.

The MOMIS FSHD Web Platform shows other different tabs, clustering patients for other features, including:

1. The number of subjects registered every year as well as the enrollment trend is a metric that is useful for planning resources and procurement.
2. Subjects enrolled by location, showing on a map the geographic distribution of the patients.
3. The distribution of alleles within subjects accrued by the INRF, on the basis of their size, for a specific phenotypic category.

All these filtering options can be combined for a finer level of analysis and the MOMIS FSHD Web Platform could then generate graphs allowing an overview of data.

4. Principle of data governance

The INRF complies with practices indicated for the good governance of a registry. The team has different and complementary expertise, including a named registry lead, a statistician, data stewardship and database management, development and hosting and a legal specialist. Each clinical center, headed by a designated candidate, has the responsibility to train their medical staff about the clinical procedures adopted by the registry. Once per year, a round table discussion is planned in order to chew over the concerns of the group and define key objectives.

With regard to privacy policies, INRF adheres to “EU General Data Protection Regulation (GDPR)”.

5. Data access

Data management is performed by DataRiver srl, which exercises the utmost care to ensure that all the confidential information stays protected from prying access.

Data is collected according to the principles of lawfulness, fairness and transparency, carefully applying specific Standard Operation Procedures (SOP) in order to prevent and detect any exposure of personal and sensitive data and adopt proper risk mitigation measures.

DataRiver follows all the best practices and the laws to ensure the protection of the personal data of the patients, all the system is compliant with EU General Data Protection Regulation (GDPR).

Data is stored on a dedicated server hosted by a UNI EN ISO 27001:2005 certified server farm to ensure the physical, logical and organizational security of information. The server is protected by firewall configurations and remote access is allowed only to the authorized system administrator through SSH connection with authentication via asymmetric key pair.

Sensitive data are stored in a database implementing encryption at the filesystem level, in compliance with the requirements of the GDPR (art.32) and with the published guidelines by the European Union Agency for Network and Information Security (ENISA—Manual on the Security of Personal Data Processing).

Data that necessary allow the identification of the subjects for research and patient’s care purposes are stored in a separate database and made available to authorized users on a role-based permission system implemented by the registry platform in order to guarantee the minimization of the access to personal data.

The access to patients’ personal information is authorized exclusively for staff working at Miogen lab, with the consensus of the curator of the Registry. Every member of the staff has personal credentials for accessing Momis platform and in agreement with the curator of the Registry have the permission to manage the data (insertion, corrections, extraction, quality control). The agreement between the curator of the Registry and the staff is to maintain the confidentiality of all the information collected. When the employment relationship is over, the access for the member of the staff is immediately interrupted. If requested, anonymized data are provided to collaborators for cohort studies.

6. Data ownership

The principal investigator of the INFR is the curator of the data and acts as the “data controller” of the registries and assumes all the responsibility for the protection of the data, its storage, use and access. When processed, the data become research data and are then the intellectual property of the investigator who is the “third party”.

7. Data management

DataRiver provides remote help-desk service and support to the users of the platform on weekdays during working hours, as established by contract. The helpdesk and support service includes the creation and management of user accounts, the solution of problems related to the ordinary platform usage and maintenance, the analysis, application and documentation of any extraordinary interventions to address issues related to the process of entry, edit and management of the registry’s clinical data. The INRF is operating to reach high-quality data level, and interoperability based on FAIR principles (i.e., Findable, Accessible, Interoperable and Reusable) in order to make data available for wider use.

## Results

### Data collection and management model for a rare disease

**Fig. 2:**
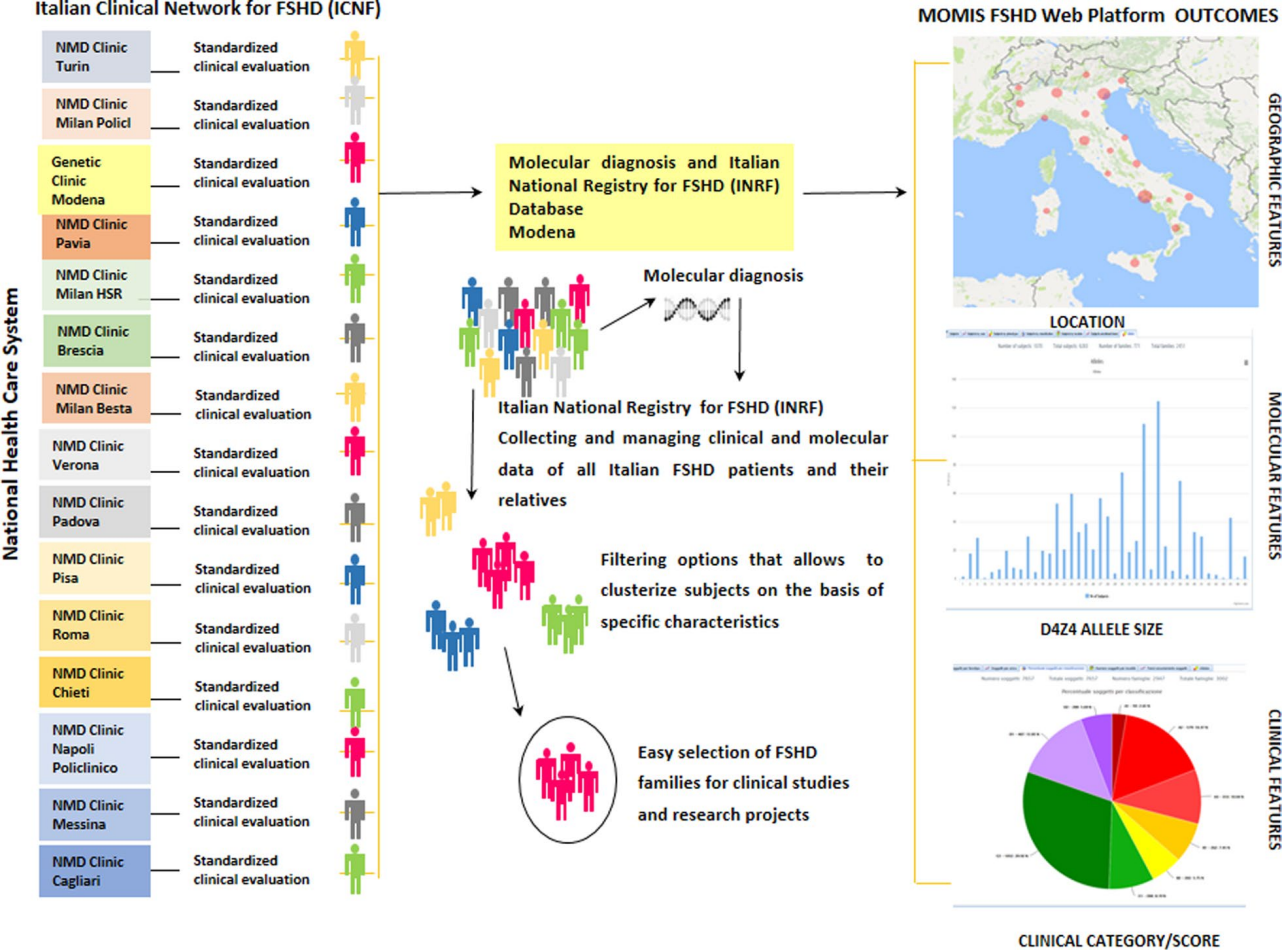
The ICNF-INRF-MOMIS FSHD Web Platform interaction network. Subjects are recruited by the ICNF, a consortium consisting of 14 centers specialized in neuromuscular diseases located across Italy. Biological samples of participants and their clinical information are collected in Modena for molecular analysis and database integration in INRF. MOMIS FSHD Web Platform filtering options allow patients selection for clinical and research purposes

The MOMIS FSHD Web Platform is the Italian national data repository for FSHD and represents a fundamental tool for collecting and managing anamnestic, clinical, and molecular data of all Italian subjects accrued through the INRF. Biological samples of participants and their clinical information were collected by the ICNF, which consists of 14 centers specialized in neuromuscular diseases located across Italy and a centralized laboratory, the Miogen lab, dedicated to molecular analyses for FSHD (Fig. 2). The Miogen lab has been a reference center for the molecular diagnosis of FSHD which has been conducted on 7197 subjects. 3362 individuals carry a DRA: 1634 probands unrelated to each other, of these 602 are isolated cases (with no other family members investigated). In 1032 cases, molecular investigation has been extended to other family members. Of 3747 molecularly tested relatives, 1728 were heterozygotes for a DRA.

Thanks to the analysis of 3577 clinical forms collected since 2009, it has been possible to establish that 2165 participants (60.5%) had muscle impairment with different degree of severity, from mild (FSHD score 1–2) to moderate (FSHD score 3–7) and severe (FSHD score 8–15). In the period 2015–2019, 2059 subjects were evaluated following the CCEF, which led to a more precise phenotypic description. Through the CCEF it was possible to demonstrate that the wide phenotypic variability of participants: (55.4%) present a myopathic phenotype compatible with FSHD (Clinical Categories A, B and D1) and 134 (6.5%) presented muscle impairment that was not compatible with FSHD diagnosis. Since the establishment of the INRF in 2007, the number of enrolled subjects has been steadily increasing until 2015, the time the CCEF has been applied for the clinical evaluation (Additional file 1A). From 2015, the enrollment of subjects with a myopathic FSHD-like phenotype (categories A, B, and D) has been steady (Additional File 1B). Since FSHD prevalence is 1 in 20,000 [1] with an estimate of 3,030 people affected by FSHD in Italy, indicate that the collection of subjects accrued by the INRF might be largely representative of the Italian population. As a result, this centralized data collection offers the possibility of conducting clinical and epidemiological studies with high statistical power.

### The MOMIS FSHD web platform application in clinical research

The most relevant feature of the MOMIS FSHD Web Platform is the population-based systematic collection of clinical and anamnestic data. This has fostered clinical and epidemiological research in a rare disease such as FSHD. It is now clear that clinical variability is a relevant part of hereditary diseases and that the disease expression and evolution is determined by several factors. In the case of FSHD this aspect is of great importance especially because the molecular marker used for FSHD diagnosis is a common polymorphism [20]. Assuming that the presence of DRA constitutes a susceptibility factor there might be several elements contributing to disease expression. In particular, the genotype–phenotype correlation on the basis of the MOMIS FSHD Web Platform’s database has generated information of medical importance either for clinical practice or for genetic counseling (Table 2). Based on our earlier consideration, it clearly appears that one of the most important usages of the MOMIS FSHD Web Platform is represented by its filtering options that allows clustering subjects based on specific characteristics. The selection of cohorts of subjects for epidemiological studies aimed at addressing specific questions is based on the MOMIS interrogation, which strongly shortens the time for the subject's collection and minimizes errors. Patients are selected based on the information reported in the database, including anamnestic information. Selection can be very specific: for example, it is possible to select people from a certain region, carrying DRA with 4–8 repeat units, who practiced sports; or we may filter for females, category A, with at least one pregnancy belonging to a family with other relatives affected. Another valuable aspect of the MOMIS FSHD Web Platform is the ability to carry out family studies, defining in each family the characteristics of the proband and relatives. As shown in Table 1, the platform has been successfully applied to many different aspects of FSHD research: 1) the CCEF validation [16] 2) the genotype/phenotype characterization of people carrying specific molecular features: 1–3 DRA [14] or 7–8 DRA [21] or 9–10 [22] 3) follow up studies [23]. The MOMIS FSHD Web Platform has also been used 4) for molecular research on D4Z4 epigenetic regulation [24] or 5) to identify genetic interactions with other rare diseases [25].

**Table 2:** Literature reports based on the use of MOMIS and OPEN CLINICA tools for the Italian FSHD Registry

Momis FSHD platform usage	Participants (n)	Study design	Conclusions
Subjects filtered by year: 2008–2013 Subjects filtered by allele: 1 - 3 RUs Subjects filtered by term: proband and relatives Subjects classified using CCEF annotations: category and FSHD score	66 probands 33 relatives	Subjects carrying 1–3 DRA were searched for disease early onset. Disease outcome was evaluated in de novo and familial probands and their relatives. To investigate the earliest signs of disease, the Infantile Anamnestic Questionnaire (IAQ) was used. Nikolic et al., 2016	The size of the D4Z4 allele is not always predictive of severe clinical outcome. The high degree of clinical variability suggests that additional factors contribute to the phenotype complexity
Subjects filtered by year: 2008–2009 Subjects filtered by allele: 1-9 RUs Subjects filtered by geographic location: Modena, Turin, and Naples Subjects classified using CCEF annotations: category and FSHD score	56 cases	Based on the 7-year experience of the ICNF, the inter-rater reproducibility of the CCEF was assessed between two examiners using kappa statistics in 56 subjects carrying the molecular marker used for FSHD diagnosis. Ricci et al., 2016	CCEF is an easy clinical tool useful to capture various phenotypes from classic FSHD to individuals with incomplete phenotype, or asymptomatic carriers as well as subjects with atypical signs for which alternative diagnoses may be supposed
Subjects filtered by year: 2008–2016 Subjects filtered by allele: 7-8 RUs Subjects filtered by term proband and relatives Subjects classified using CCEF annotations: category and FSHD score	187 probands 235 relatives	Probands and their relatives carrying 7–8 DRA were phenotypically stratified based on the CCEF categories. Ruggiero et al., 2020	Large phenotypic variability was found in individuals carrying a 7–8 DRA, in contrast to the indication that a positive molecular test is the only determining aspect for FSHD diagnosis. Carriers of a 7–8 DRA constitute a genetic subgroup different from classic FSHD. The use of CCEF and the family study are both required for clinical management and genetic counseling
Subjects filtered by year: 2008–2016 Subjects filtered by allele: 9-10 RUs Subjects filtered by term: proband and relatives Subjects classified using CCEF annotations: category and FSHD score	134 probands 110 relatives	Probands and their relatives carrying 9 to 10 DRA were phenotypically stratified based on the CCEF categories. Ricci et al., 2020	Large phenotypic variability in 9–10 DRA carriers, including 70% of healthy relatives. Penetrance in families ranged between 20–100%. The use of CCEF and the family study are both required for clinical management and genetic counseling. Stratification of patients using the number of D4Z4 RUs is not accurate
Subjects filtered by term proband and relatives Subjects classified using CCEF annotations: Category and FSHD score Disease progression measured as ΔFSHD score	141 probands 105 relatives	Subjects were analyzed to estimate the disease worsening calculated as the FSHD score performed at baseline and at the end of 5-year follow-up (ΔFSHD score) Vercelli et al., 2020	The progression of disease is different between index cases and carrier relatives and the assessment of the CCEF categories has strong prognostic effect in FSHD1 patients
Subject filtered by clinical category: A1–A3, FSHD score > 1 Subjects filtered by term: proband and relatives Subjects (relatives) classified using CCEF annotations: category and FSHD score	122 probands 110 relatives	The D4Z4 methylation level at 4q35 was assessed in 122 FSHD1 probands and in relatives carrying the same molecular defect presenting classical FSHD (category A), or incomplete /complex phenotype (categories B or D) or no muscle impairment (category C). Nikolic et al., 2020	The D4Z4 methylation levels among index cases and in families show a high variability with no association with clinical manifestation or disease progression The results of D4Z4 methylation analysis must be cautiously interpreted in respect to disease prognosis, which requires family studies
Subjects filtered by term proband Subjects filtered for non-familial cases Subjects filtered by comorbidities/complex phenotypes	1339 FSHD unrelated cases	Among 1339 unrelated FSHD cases 3 unrelated cases presented signs of Williams-Beuren Syndrome (WBS) in early childhood and later developed FSHD. All 3 cases carry both molecular defects associated with the two disorders. Rodolico et al., 2020	The rarity of WBS and FSHD, 1 in 7500 and 1 in 20,000 respectively, argued for a nonrandom association of the two diseases. These cases open novel and unexpected interpretation of genetic findings providing hints for the identification of genes and functional pathways involved

### INRF in the scheme of the world

Several entities for patients’ data collection have been established in the last years including national, European and global Rare Diseases (RDs) registries responding to the rising requests from patient organizations, regulatory agencies and healthcare professionals for curated data serving epidemiological and clinical research on RDs.

At the European level, several platforms have been create: the European Platform for rare disease registries (EPIRARE: www.epirare.eu), the International Rare Disease Research Consortium, (IRDiRC: www.irdirc.org) and the Integrated Platform connecting databases, registries, biobanks and clinical bioinformatics for Rare Disease research (RD-Connect: www.rd-connect.eu). In February 2017, 24 European Reference Networks (ERNs) (https://www.ern-rnd.eu) were established in a European legal framework, of which 23 are dedicated to rare or low prevalence complex diseases or conditions. An Italian national institutional registry of rare disease patients, the *Registro Nazionale Malattie Rare* (RNMR), has been established in Italy in 2001 by the National Centre for Rare Diseases (*Centro Nazionale Malattie Rare*, CNMR) of the *Istituto Superiore di Sanità* (National Institute of Health) [26]. The registry is not exhaustive of all RDs and FSHD is underrepresented as well as other Neuromuscular disorders (NMD) as this database comprises RDs characterized by congenital syndromes, severe clinical conditions, high level of invalidity, and costly treatments. Here, diseases of the musculoskeletal system and connective tissue are globally the 5.5% of total records.

Neuromuscular diseases specific registries are present worldwide and globally described within the TREAT-NMD neuromuscular network website (https://treat-nmd.org/patient-registries). The TREAT-NMD network has developed a specific tool, the TREAT-NMD Global Registry Network, where registries are listed regarding the principal NMD disorders worldwide, including FSHD. Recently, a collaboration between TREAT-NMD and ERNs was launched, leading to the establishment of a **“**FSHD European Trial Network”. The INRF is part of this network.

**Fig. 3:**
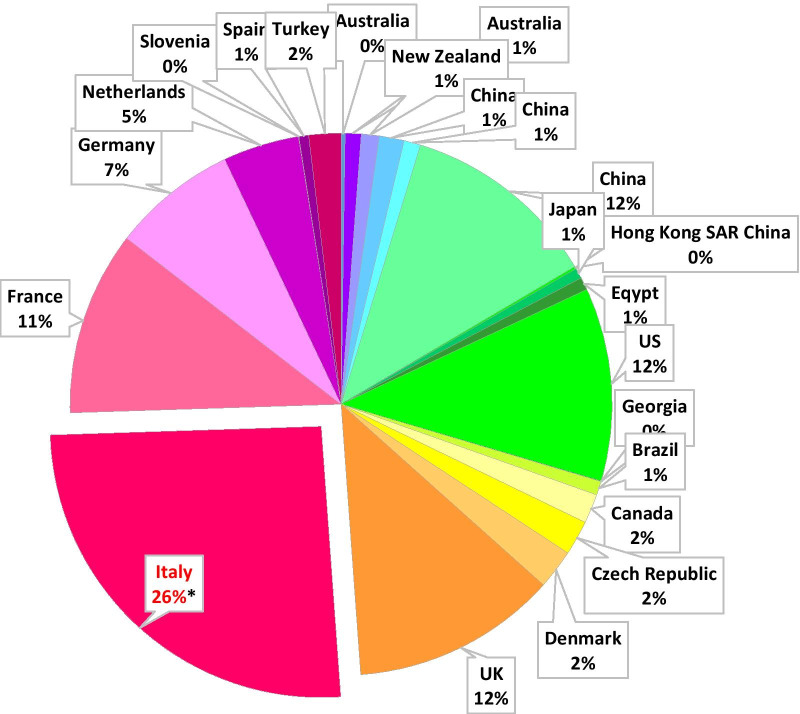
The INRF in the scheme of the world. Pie chart showing the percentage value of FSHD subjects present in each FSHD registry worldwide over the total number of patients accrued by all registries. *Data updated to august 2021

According to data provided by the FSH Society, we were able to compare a total of 23 FSHD registries located in 5 continents (4 in Asia, 1 in Africa, 4 in America, 11 in Europe and 3 in Oceania) [27–32] which were collectively analyzed based on patient’s number within and on specific features as reported in Fig. 3 and in Additional file 2. In particular, Fig. 3 shows the number of FSHD subjects present in each platform as the percentage value over the total number of patients accrued by all registries. Notably, the pie chart highlights that the INFR alone accounts for 26% of total FSHD patients worldwide. Given the reported values, we selected registries including a significant number of patients, at least 5% of total number, for further consideration. Additional file 2 shows demographic, clinical/molecular and logistics parameters. We also evaluated the ratio between the number of accrued FSHD subjects over the total country population. This parameter varies among countries and may be useful to estimate the ‘recruitment power’ of single registries. Additional file 2 also shows that the INFR platform is unique among the various FSHD registries, as clinical and anamnestic information are totally reported by the ICNF-clinicians through the CCEF template and all accrued subjects are molecularly investigated.

## Discussion

The expanding use of DNA molecular analysis applied to diseases with a genetic basis has revealed increasingly varied phenotypic spectra in patients carrying mutations in the same gene, as one can see by the number of reviews [3, 33, 34].

It is now clear that the well-phenotyped and genetically characterized patient cohorts can promote a more accurate treatment of patients. These steps are necessary to develop appropriate outcome measures and biomarkers and to proceed towards trial readiness. The limited knowledge about the natural history of RDs, their chronicity, need for long-term follow-up and high demand for assistance represent a special challenge for the healthcare systems. The management of all these needs and questions requires a large amount of information to be collected. In this respect RD or disease-specific data collection hubs with the possibility of integrating data available in different locations and the management of continuously increasing information related to a single sample are key instruments for the surveillance of these diseases, with the aim of improving patients and caregivers quality of life and for therapeutic planning. Thus, the term "Registry" has now gone beyond the original significance of a collection of mere anagraphic/demographic data. The great effort is directed towards combining genomic, anamnestic and clinical investigation. This important process requires a solid description of clinical data and appropriate Big Data techniques to exploit the potential of the wealth of information that can be acquired. In the case of FSHD, the MOMIS FSHD Web Platform serves these purposes. The collection of participants evaluated with a standardized methodology with demonstrated interrater reliability is the major strength of the INRF and the platform implemented for INRF data management based on MOMIS and OpenClinica is a solid foundation both for the secure users access to individual’s data and for the clinical management of patients. This structure fosters the definition of epidemiology of FSHD as well as definition of its natural history, two elements that are crucial for the design of therapeutic interventions.

A mid-term goal of the INRF is the identification of factors, including comorbidities or lifestyle habits that might influence the disease onset and progression. These might be identified through epidemiology studies based on the analysis of anamnestic records of a vast number of subjects and pedigrees. Moreover, the collection of precisely phenotyped participants might permit their stratification on the basis of standardized criteria, providing the framework for further studies that might serve clinical practice and basic research in a rare, understudied disease.

The FSHD registry is an evolving tool: this is one of its most important features in order to allow longitudinal studies and increasing amount of data elaboration, but it represents a double edged sword as the registry requires to be constantly updated with follow-up information of patients to fully exploit its function, otherwise it is in danger for becoming a statical survey of a fixed cohort, which is not our purpose. It would be of essential help to look for an implementation in our database which could facilitate the fill in of individual clinical records both for clinicians and patients themselves.

In the future, Patient Engagement and Mobile data capture solutions can be integrated into the existing database to empower patients through self-assessment of health indicators. Wearable and clinical devices can be used to automatically collect physiological data. Innovative communication strategies will also improve the patient’s engagement and Patient Reported Outcomes data (ePRO) can be collected in real time employing the patient’s smartphones and mobile devices.

To this purpose, it would be of essential help in the future to look for an implementation in our database which could facilitate the fill in of subject’s clinical records both for clinicians and patients themselves.

Our experience with the INRF shows the possibility of the systematic collection of clinical data for rare genetic diseases on a national scale and its value is providing informative observational data that can support clinicians, researchers and the health system in acquiring precise information on rare disorders. We are confident that our experience with the integrated management of FSHD patients would be taken as an example and applied to other complex diseases in which the genotype/phenotype correlation is far to be resolved.

## Conclusion

The future of medicine lies in personalized treatments. In the case of rare genetic diseases, the integration of data from genomic analyses, standardized clinical evaluation, collection of environmental and lifestyle data, is a precondition to implement person-centered care. The systematic collection of clinical data for rare genetic diseases on a National scale is the tool providing informative observational data that can support this process.

We propose our experience with the integrated management of data in a rare disease such as FSHD as a white label that could be applied to other complex diseases in which the genotype/phenotype correlation is far to be resolved.

## Supplementary Information

**Additional file 1**. Trends in enrolment of patients collected by INRF. (A) Distribution of clinical categories as described by the CCEF within subjects with a myopathic FSHD-like phenotype since the start of the CCEF utilization (2015-2019) (B) Graphic representation of the INRF enrolment in the period 2007 to 2019. The annual number of subjects is reported.

**Additional file 2**. Summary of main FSHD-registries features.

## Data Availability

Please contact author for data requests. To date, the INRF is not an open access database. The stored data have been available for the Italian Clinical Network for FSHD (ICNF) since its establishment. The Miogen Lab welcomes any researcher focused on FSHD or different genetic diseases to make contact for collaborative project proposals. Applications for Access to the INRF data may be directed (providing the corresponding documentation required) to www.FSHD.it (managed by Miogen Lab at University of Modena and Reggio Emilia) and should contain a brief description of the related research project.

## Abbreviations

FSHD: Facioscapulohumeral dystrophy
DRA: D4Z4 reduced allele
PAS: Polyadenilation signal
CCEF: Comprehensive clinical evaluation form
INRF: Italian National Registry for FSHD
ICNF: Italian Clinical Network for FSHD
MOMIS: Mediator Environment for Multiple Information Sources
DB: Database
EDC: Electronic data capture
