# Supplementary figures and images for: The Italian National Registry for FSHD: an enhanced data integration and an analytics framework towards Smart Health Care and Precision Medicine for a rare disease
Source: Orphanet J Rare Dis. 2021 Nov 4;16:470. doi: 10.1186/s13023-021-02100-z (PMC8567605; doi:10.1186/s13023-021-02100-z)

A

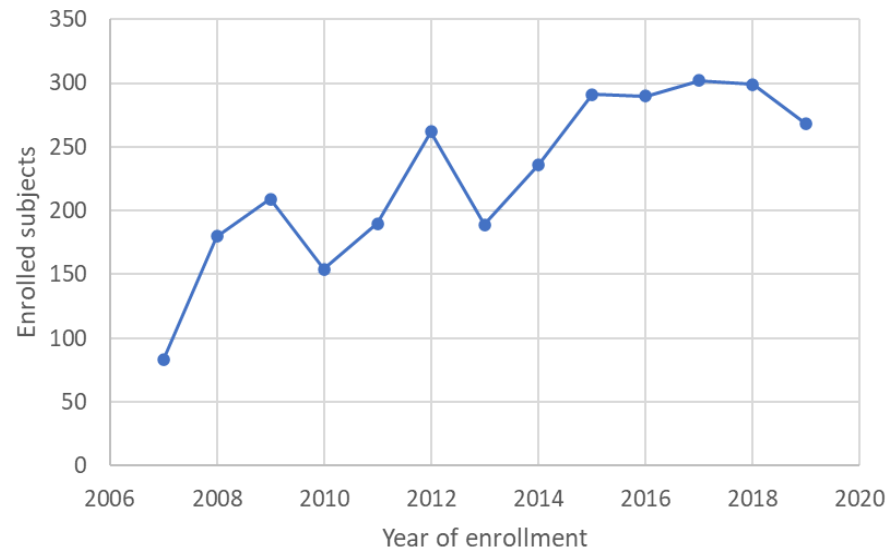

B

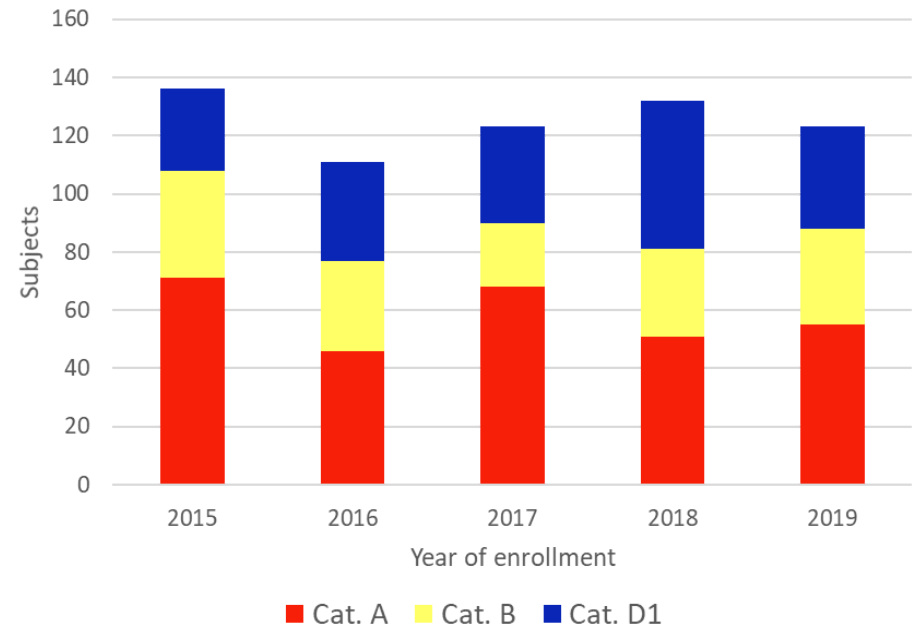

Additional File 1

Supplement: Supplementary file 1 — Additional file 1. Trends in enrolment of patients collected by INRF. (A) Distribution of clinical categories as described by the CCEF within subjects with a myopathic FSHD-like phenotype since the start of the CCEF utilization (2015-2019) (B) Graphic representation of the INRF enrolment in the period 2007 to 2019. The annual number of subjects is reported. [file 13023_2021_2100_MOESM1_ESM.pdf]
