# Supplementary material for: The Italian National Registry for FSHD: an enhanced data integration and an analytics framework towards Smart Health Care and Precision Medicine for a rare disease
Source: Orphanet J Rare Dis. 2021 Nov 4;16:470. doi: 10.1186/s13023-021-02100-z (PMC8567605; doi:10.1186/s13023-021-02100-z)
Supplement: Supplementary file 2 — Additional file 2. Summary of main FSHD-registries features. [file 13023_2021_2100_MOESM2_ESM.pdf]

Summary of the principal FSHD registries features-updated to 2021							
**Registry Country**	**Italy**	**UK**	**China**	**US**	**France**	**Germany**	**Netherlands**
**Registry name**	Italian National Registry for FSHD (INRF)	UK FSHD Patient Registry	Chinese Clinical Trial Registry: Registration study of FSHD	National Registry of Myotonic Dystrophy and FSHD Patients and Family Members	The French National Registry of Facioscapulohumeral Dystrophy	German National FSHD Registry	FSHD registratie
**Scale of the registry**	National	National	Regional	National	National	National	National
**Date of establishment**	2007	2013	2020	2000	2013	2017	2015
Total of FSHD Patients	***2.169****	***1.035***	***998***	***975***	***930***	***630***	***384***
Country population	***59.258.000***	***68.168.033***	***1.443.615.491***	***332.915.073***	***65.426.176***	***83.995.966***	***17. 550.654***
Recruiting power	***1:27.000***	***1:65.862***	***1: 1.446.508***	***1:341.451***	***1:70.000***	***1:133.326***	***1:45.704***
**Data collection**	Clinical assessment, patient reported, genetic tests, family histories, Clinical categorization based on phenotypic scores and features developed by the Italian Clinical Network for FSHD	Patient reported (online), genetic tests, family histories, motor function assessment	Clinical assessment, patient reported, genetic tests, family histories, motor function assessment	Patient reported (online), genetic tests, family histories, motor function assessment	Clinical assessment, patient reported, genetic tests, family histories,	Clinical assessment, patient reported, genetic tests, family histories,	Clinical assessment, patient reported, genetic tests, family histories,
**FSHD-Specific tool for clinical evaluation**	Yes<br>Comprehensive Clinical Evaluation Form (CCEF)	Yes<br>FSHD Pain questionnaire	No	No	Yes<br>Clinical Evaluation Form (CEF)	No	No
**Longitudinal studies**	Yes	Yes	No	Yes	Yes	Yes	Yes
Genetic diagnosis required	***Yes***	***No***	***Yes***	***No***	***Yes***	***No***	***No***
Participants with genetic diagnosis	***100%***	***50%***	***100%***	***57%***	***96%***	***51%***	***100%***
Data reports from	***Clinicians***	***Patients***	***Clinicians and Patients***	***Clinicians and Patients***	***Clinicians and Patients***	***Patients***	***Patients***
**Access to data**	Yes<br>upon request<br>No fee	Yes<br>upon request<br>Usage Fee	Yes<br>upon request<br>No fee	Yes<br>upon request<br>No fee	Yes<br>upon request<br>Usage Fee	Yes<br>Upon request<br>No fee	Yes<br>upon request<br>No fee
**Feedback to Patients-Divulgative**	Yes<br>FSHD day on a yearly basis. Webinar on specific topics	No	No	Sporadic newsletters	Sporadic newsletters	Sporadic newsletters	No
**Founds**	Non-profit grants	Non-profit grants form patients association	Non-profit grants	Non-profit grants	Non-profit grants	Non-profit grants	Non-profit grants

**Data updated to august 2021*

Additional File 2
